# Interval Optimization Model Considering Terrestrial Ecological Impacts for Water Rights Transfer from Agriculture to Industry in Ningxia, China

**DOI:** 10.1038/s41598-017-02734-9

**Published:** 2017-06-14

**Authors:** Lian Sun, Chunhui Li, Yanpeng Cai, Xuan Wang

**Affiliations:** 1 Key Laboratory for Water and Sediment Sciences of Ministry of Education, School of Environment, Beijing Normal University, Beijing, 100875 China; 2 Beijing Engineering Research Center for Watershed Environmental Restoration & Integrated Ecological Regulation, School of Environment, Beijing Normal University, Beijing, 100875 China; 3 State Key Laboratory of Water Environment Simulation, Beijing Normal University, Beijing, 100875 China

## Abstract

In this study, an interval optimization model is developed to maximize the benefits of a water rights transfer system that comprises industry and agriculture sectors in the Ningxia Hui Autonomous Region in China. The model is subjected to a number of constraints including water saving potential from agriculture and ecological groundwater levels. Ecological groundwater levels serve as performance indicators of terrestrial ecology. The interval method is applied to present the uncertainty of parameters in the model. Two scenarios regarding dual industrial development targets (planned and unplanned ones) are used to investigate the difference in potential benefits of water rights transfer. Runoff of the Yellow River as the source of water rights fluctuates significantly in different years. Thus, compensation fees for agriculture are calculated to reflect the influence of differences in the runoff. Results show that there are more available water rights to transfer for industrial development. The benefits are considerable but unbalanced between buyers and sellers. The government should establish a water market that is freer and promote the interest of agriculture and farmers. Though there has been some success of water rights transfer, the ecological impacts and the relationship between sellers and buyers require additional studies.

## Introduction

In many places across the world, decision makers or water managers are normally facing a dilemma to supply sufficient water to agricultural irrigation and to meet increasing water demand by industrial development^1^. Such competitions over limited water resources from agriculture and industry sectors have resulted in many water scarcities. At the same time, public concerns and objections to increase water withdrawals and exploitation of extra water sources is growing^2^. Thus, effective tools to support optimal allocation of limited water resources among multiple sectors are desired. Considering the cap and trade system of water, transferring and trading water rights is considered an attractive tool of water resources management to address water scarcity^3, 4^.

Transferring and trading water rights could generate significant externalities including ecological or environmental effects^5, 6^. Many researchers have studied the influence on surface and groundwater flow regimes resulting from trading water rights^5, 7–9^. Other studies analyzed the impacts of trading on aquatic ecology, most of which evaluated effects on environmental flow^10–14^. Some studies assessed the comprehensive effects of water trading on aquatic ecosystems^9^. However, most studies have focused on aquatic but not terrestrial ecology. Water transfer, especially within the agriculture sector, is closely related to irrigational ecosystems including both farmland and natural ecosystems within the irrigation area. The trading of water rights can change the water regime in irrigation area, thus affecting the irrigational ecosystem. Therefore, the impacts of water rights transfer between sectors on terrestrial ecology should be examined.

China’s first pilot project of water rights transfer was implemented in 2004 in the Ningxia Hui Autonomous Region (referred here as Ningxia), which aimed to alleviate the situation of excessive water usage by transferring water rights from agriculture to industry^3, 15^. Since then, there has been study of the effective allocation of water rights between different sectors^16, 17^. The quantity of transferable water is determined based on the calculation of water-saving potential from agriculture engineering measures^18, 19^. However, since the quantity of optimal water is still unknown, the water saving potential was actually an upper threshold for the water that was transferred. Some research used multi-objective programming to allocate the water rights of society, ecology, and industry in Ningxia^20^. Nevertheless as the two major sectors in water rights transfer, the optimal allocation of water between agriculture and industry remains unclear. Furthermore, these transfers are obviously constrained by economic benefits, but the limitation from ecology has largely been ignored^21^. Applying the constraints of ecology would alter the optimal allocation regime. Therefore it is essential to determine the optimal water allocation for a water rights transfer system in Ningxia.

The process of determining the volume of transferable water rights are filled with uncertainties. Many parameters (e.g., water quota of irrigation, conversion coefficient of saved water from diversion to consumption, and water demand of industrial enterprises *et al*.) fluctuate in their intervals^22, 23^. Hence, at present the accounting of transferable water is a kind of precise calculation with fixed value^18, 24^. Therefore, it is essential to use interval method to study water rights transfer from agriculture to industry. Besides, the uncertainty comes from the Yellow River. As the source water of transferring water rights, the runoff of the Yellow River varies apparently years^25^. If the same volumes are still transferred in drought year, agriculture would suffer lost, which would decrease the benefits of water rights transfer system. Therefore to compensate agriculture is indispensable to reflect the fluctuation of the runoff.

In this study, an interval optimization model is proposed to maximize the benefits of a water rights transfer system that includes industrial and agricultural sectors in Ningxia. The model is subjected to a number of constraints including the water saving potential from agriculture and ecological impacts. The terrestrial ecological impacts in irrigation area will be investigated based on ecological water levels that serve as indicators of surface vegetation growth. The interval method will be applied to address the uncertainty of parameters. Two scenarios will be used to study the difference in potential benefits of water rights transfer. The runoff of the Yellow River, the source water of the transfer, varies significantly in volume in different years. Altered frequencies may affect the production of agriculture, and compensation for agriculture will be considered.

In 2016, the Ministry of Water Resources approved the management regulation of water trading to guide a national water market, and water trading including water rights transfer will be established in additional regions in China^26^. Therefore this study not only establishes a water rights transfer model that considers ecological impacts, but also provides meaningful guidance for continued development of future water trading programs in China.

## Results

### The thresholds of water rights transfer

**Figure 1:**
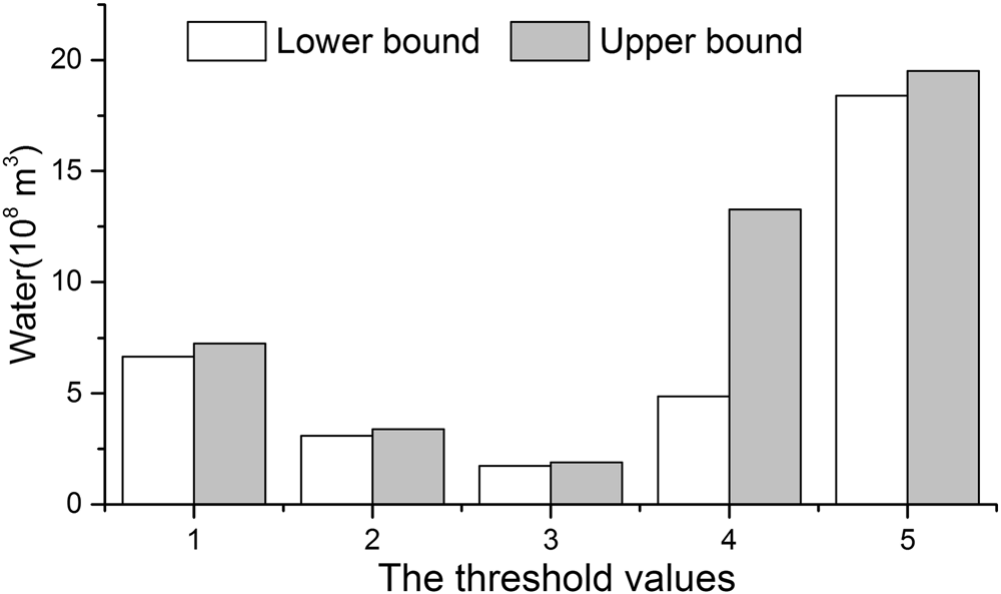
The saving and threshold of water rights transfer. Groups on the coordinate axis: 1 represent the threshold value of water saving from canal lining, 2 are from structural adjustment, and 3 are from drip irrigation, respectively; 4 represent the threshold values of best conditions for natural vegetation growth, and 5 are the values for desertification respectively.

The canal system was generalized using the equal canal system method. Then combined with the volumes of diverted water of each sub-irrigation area (Table 1), the thresholds of water saving from canal lining were calculated (Fig. 1).

**Table 1:** Current state of the canal system in the NIA.

Canal (grade)	Sub-irrigation area	Length/km	Lining length before 2004/km	Lining length after 2004/km	The rate of canal lining
Head main canals(1)	Hexi	47.1	3.4	/	7.2%
	Hedong	5	0.8	/	16.0%
	Weining	/	/	/	/
Main canals(2)	Hexi	596	56.2	439.6	83.2%
	Hedong	205.1	48	108.6	76.3%
	Weining	332	87.7	106.9	58.6%
Branch canals(3)	Hexi	3767.2	1069.2	1903.6	78.9%
	Hedong	1396.5	251	247	35.7%
	Weining	1827.4	159.3	463.5	34.1%
Lateral canals(4)	Hexi	3790.7	352.5	855.2	31.9%
	Hedong	768	315.1	110.9	55.5%
	Weining	995.3	58.2	208.3	26.8%
Field ditches(5)	Hexi	17538.7	476.3	/	2.7%
	Hedong	5852.9	190.9	/	3.3%
	Weining	2645	61.4	/	2.3%
Sum	/	39766.9	3130	4443.5	19.0%

In 2004, the local governments of Ningxia implemented the water rights transfer through canal lining.

The three main crops (spring wheat, maize and paddy) dominate the NIA which occupies 89% area^27^. As a consequence, the saved water from structural adjustment of crop plantation is focused on these three crops. Since the net irrigation water quota of these three crops are different, the maximum saved water when all the lands are planted with maize will have the least water demand. Based on equation (5), the threshold of structural adjustment for wheat and paddy is shown in Fig. 1. In terms of the amount of saved water from using drip irrigation technology, having all the land covered with drip irrigation technology would produce the largest saving potential. Thus, based on the net irrigation water quota after using drip technology for wheat and maize, the threshold of the water saving would be reached (Fig. 1).

As described in the methods in the supplementary information, the hydrogeological parameters could be determined based on the specific water depths in Table 2. Combining the geographic data of the sub-irrigation area (i.e., area, precipitation, evapotranspiration capacity, and water depth) with equation (16), the different amounts of water intaken from the Yellow River could be calculated. Then the difference of the amount of water intaken between current water depth and ecological targets represents the corresponding saved water. Since the current average water depths of each sub-irrigation area (2.10 m) are deeper than the depth of the ecological targets (salinization, moisture situation in root zones, the largest output for corn, the largest output for crops) in Table 2, therefore the target of desertification and best growing for natural vegetation are selected as the critical ecological factors.

**Table 2:** Groundwater depths for ecological targets.

Ecological target	Depth (m)	Value attribution	Source
Salinization	0.86	Upper depth	Shang^28^
Moisture situation in root zones	1.3	Upper depth	Hao *et al*.^29^
Desertification	6.2	Lower depth	Jin *et al*.^30^
Best conditions for natural vegetation growth	3.0~3.5	Interval depth	Jin *et al*.^30^, Zhang and Huang^19^
The largest output for corn	1.4~1.55	Interval depth	Hao *et al*.^31^
The largest output for crops	1.85	Single depth	Zhang *et al*.^32^

The total water saving potential *WD* would be 34.86 × 10^8^ m^3^, and in these two ecological targets, the threshold of ecological impacts $W{T}_{e}^{\pm }$ might be set as the large value: [18.40, 19.51] × 10^8^ m^3^ (grouped column 5 of Fig. 1).

### The results of interval optimization model

The length of the head main canals in the Hexi and Hedong sub-irrigation area are short, and there is no head main canal in the Weining sub-irrigation area (Table 1). Therefore in the interval model we assumed that all the head main canals would be lined. In other word, *x*_11_ = 1, *x*_21_ = 1. The price of agricultural water *R*_*w*_ now is 0.071 yuan/m^3^.

Two scenarios were used to reflect the actual and potential values of water rights transfer. The water demand of the future 51 coal chemical projects is [4.24, 5.44] × 10^8^ m^3^. In the planned scenario, transferable water is subjected to this demand; in the unplanned scenario, this constraint is removed. All the objective function and other constraint conditions remain the same for these two scenarios. The solutions of the interval model of these two scenarios are as follows.

For the planned scenario:

$${x}_{1}^{\pm }=0$$, $${x}_{2}^{\pm }=1$$, $${x}_{3}^{\pm }=0$$, $${x}_{4}^{\pm }=0$$, $${x}_{5}^{\pm }=0$$, $${x}_{12}^{\pm }=0.832$$, $${x}_{13}^{\pm }=0.789$$, $${x}_{14}^{\pm }=[0.319,1]$$, $${x}_{15}^{\pm }=[0.154,0.287]$$, $${x}_{22}^{\pm }=0.763$$, $${x}_{23}^{\pm }=0.357$$, $${x}_{24}^{\pm }=1$$, $${x}_{25}^{\pm }=[0.176,0.220]$$, $${x}_{32}^{\pm }=0.586$$, $${x}_{33}^{\pm }=0.341$$, $${x}_{34}^{\pm }=[0.286,1]$$, $${x}_{35}^{\pm }=[0.883,1]$$

For the unplanned scenario:

$${x}_{1}^{\pm }=0$$, $${x}_{2}^{\pm }=1$$, $${x}_{3}^{\pm }=0$$, $${x}_{4}^{\pm }=0$$, $${x}_{5}^{\pm }=[0.828,0.955]$$, $${x}_{12}^{\pm }=0.832$$, $${x}_{13}^{\pm }=0.789$$, $${x}_{14}^{\pm }=[0.733,1]$$, $${x}_{15}^{\pm }=[0.463,0.535]$$, $${x}_{22}^{\pm }=0.763$$, $${x}_{23}^{\pm }=0.357$$, $${x}_{24}^{\pm }=1$$, $${x}_{25}^{\pm }=[0.491,0.522]$$, $${x}_{32}^{\pm }=0.586$$, $${x}_{33}^{\pm }=0.341$$, $${x}_{34}^{\pm }=1$$, $${x}_{35}^{\pm }=1$$

Thus, the main water saving measures will be adjusted and the benefits will be changed correspondently (Table 3).

**Table 3:** Saved water and benefits of water rights transfer system.

Type (Unit)	Variable	Symbol	Values of planned scenario	Values of unplanned scenario
Saved water (10^8^ m^3^)	Saved water from canal lining	*WS* ^±^	[6.30, 8.32]	20.15
	Saved water from structural adjustment	*WP* ^±^	[6.55, 6.79]	[6.55, 6.79]
	Saved water from using drip irrigation	*WB* ^±^	0	5.29
	Total consumptive water saved	*WT* ^±^	[4.24, 5.44]	[10.55, 11.60]
Benefits (10^8^ yuan)	Benefit of water rights transfer system	*F* ^±^(*X*)	[660.5, 1775.6]	[1558.6, 3729.3]
	Benefits of agriculture	*F*_1_^±^(*X*)	[28.9, 44.1]	[31.8, 50.2]
	Income of structural adjustment	*f*_*c*_^±^(*X*)	[27.8, 42.9]	[27.8, 42.9]
	Income of selling the saved water from structural adjustment	*f*_*p*_^±^(*X*)	[1.12, 1.16]	[1.12, 1.16]
	Benefit of increased production as a result of drip irrigation	*f*_*r*_^±^(*X*)	0	[2.9, 6.1]
	Benefits of coal chemical industry	*F*_2_^±^(*X*)	[496.3, 1348.9]	[1526.8, 3679.2]
	Benefits of selling the production of coal chemistry	*f*_*d*_^±^(*X*)	[642.9, 1737.0]	[1600.5, 3705.3]
	Expenditure of investing in the water saving engineering	*f*_*w*_^±^(*X*)	[4.3, 10.1]	[25.0, 72.6]

### The compensation to agriculture

The water guarantee of industry (95%) is higher than that of agriculture (75%) in Ningxia. The optimization calculation is based on the annual average runoff (about 50%). Thus, when the frequency of runoff of the Yellow River is more than 50%, it is necessary to compensate for the loss of agriculture. When the frequency of runoff is equal to or less than 50%, we assumed that the water of agriculture is sufficient and does not require compensation.

**Figure 2:**
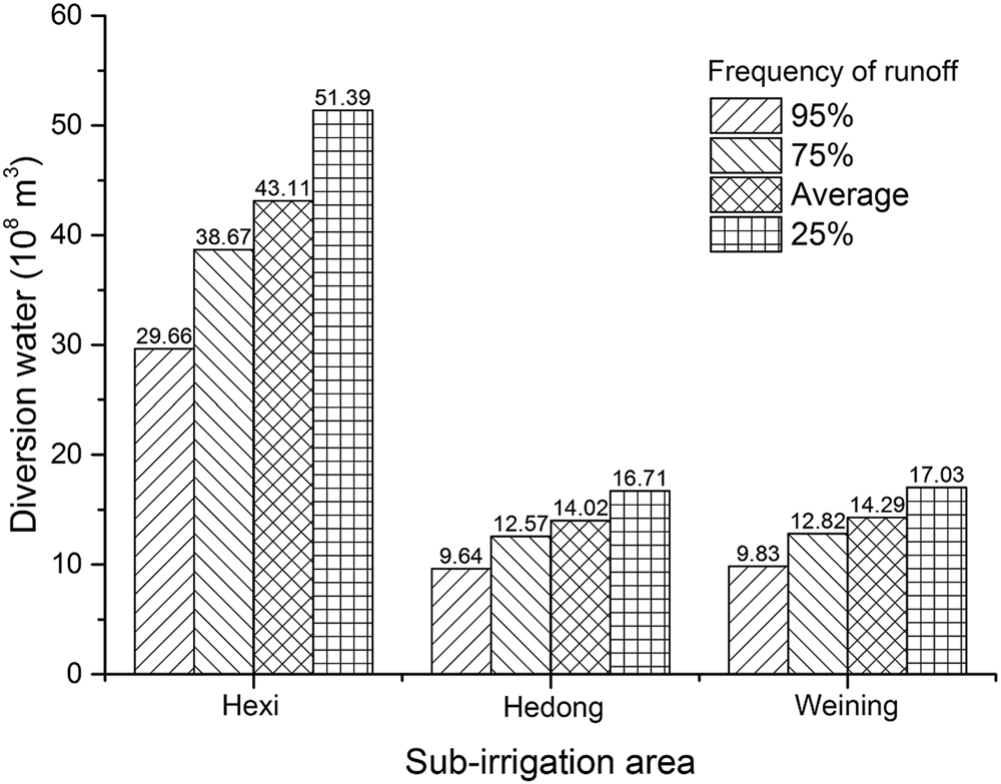
The amount of diverted water at different frequencies for three areas. Data are from Ningxia Water Conservancy^18^.

According to the optimization results, the land in the NIA is planted with maize but no wheat or paddy. The production of maize will be increased 11% when there is enough irrigation water compared with the situation without irrigation, which is equivalent to an increase of [72, 101] yuan per unit area. The synthetical net irrigation water quota is equal to the general water quota ${m}_{2}^{\pm }=[270,290]$. Therefore, based on equation (43) and the water allocation in the different runoff frequencies (Fig. 2), the compensation fees can be calculated. For the planned scenario, the compensation would be [3.19, 6.35] × 10^7^ and [1.05, 2.10] × 10^7^ yuan when the frequencies of runoff are 95% and 75% respectively. For the unplanned scenario, the compensation would be [7.93, 13.54] × 10^7^ and [2.62, 4.47] × 10^7^ yuan when the frequencies of runoff are still 95% and 75%, so the compensations of the unplanned scenario are more than twice the planned scenario, and in the same scenario, the compensations of 95% frequency of runoff are more than three times that for 75%.

## Discussion

According to the results, more water can be saved and more water rights can be transferred. Based on our model, the saved consumption water (*WT*) and the corresponding saved diverted water (*WD*) of planned scenario are [4.24, 5.44] × 10^8^ m^3^ and [11.78, 16.84] × 10^8^ m^3^ respectively. In 2009, Zhang *et al*. calculated the appropriate water-saving potential of the NIA, in which the ecological impacts were also taken into consideration^32^. Their results showed the NIA had 22.31 × 10^8^ m^3^ saved diverted water when the water depth was 1.62 m at that time. If we remove the saved diverted water of 7.99 × 10^8^ m^3^ when water depth decreases from 1.62 m to 2.10 m (the current one), Zhang’s value would be 14.32 × 10^8^ m^3^ which is in the interval we calculated. Therefore, the proposed optimized model is reasonable. More water can be transferred and more benefits can be achieved for unplanned scenario. In reality, the water rights transfer system has additional volumes of [5.11, 7.36] × 10^8^ m^3^, and has another system benefits up to 3068.8 × 10^8^ yuan (i.e., the difference between the two scenarios). This would be a positive signal for local economic development because it indicates that additional coal chemical or other enterprises could be established by transferring water rights from agriculture.

Based on the results of the optimization model, we can see that not all constraints take effect in this water rights transfer case of NIA. The volumes of transferable water are subjected to the water demand of the coal chemical enterprises [4.24, 5.44] × 10^8^ m^3^, the total water saving potential [11.49, 12.53] × 10^8^ m^3^, and the ecological impact [18.40, 19.51] × 10^8^ m^3^. The ecological impacts are not effective constraints in either scenario. This may be because the water depth of anti-desertification (6.2 m) is deeper than the current groundwater depth (2.10 m) and other ecological targets (0.86~3.5 m), which relaxes the range of constraints. However this may become a critical constraint in locations where the water depth of ecological targets are not deeper or where there is not available space to decrease the water depth. For example, if the target of *best conditions for natural vegetation growth* (3.0~3.5 m) is used as the ecological constraint, it can take effect. Since the interval value of this target ([4.87, 13.26] × 10^8^ m^3^) is partly less than the total water saving and the water demand of coal chemical enterprises. Therefore, the ecological impacts should still be a focus.

Although water rights transfer can produce enormous benefits, the benefits are imbalanced for different sectors. In Ningxia the established coal chemical enterprises have achieved a benefit of 525.3 × 10^8^ yuan in the year of 2013^22^. If all the 51 future coal chemical enterprises are established, there would be an additional [496.3, 1348.9] × 10^8^ yuan benefit according to the results of the planned scenario. The benefits from the unplanned scenario are much more. However, in terms of agriculture, the prospect is not as attractive. Currently, the benefit of agriculture in the NIA is 30.5 × 10^8^ yuan^22^. In the planned scenario, the total benefit of agriculture is [28.9, 44.1] × 10^8^ yuan. Even in the unplanned scenario, this number rises only to [31.8, 50.2] × 10^8^ yuan. Therefore agriculture does not benefit as much from water rights transfer especially compared with coal chemical enterprises.

Consequently, in water rights transfer in the NIA, the agriculture sector should be of greater focus. The structural adjustment has met its potential according to the optimization results (*x*_1_ = 0, *x*_2_ = 1, *x*_3_ = 0), but the income from selling the saved water from structural adjustment ([1.12, 1.16] × 10^8^ yuan) contributes only a little of the total benefit of agriculture. This situation is related to the relatively low water prices regulated by local government (0.071yuan/m^3^). The drip irrigation technology may create considerable benefit ([2.9, 6.1] × 10^8^ yuan), which suggests a favorable prospect to increase the benefit of agriculture especially in the situation that the low level usage of the technology currently^19^. In fact, the use of drip irrigation technology contributes most of the increasing benefit moving from the planned scenario to the unplanned scenario.

Similarly, the status of farmers should also be a main focus. On the one hand, farmers would suffer from the risk of scarcity of irrigating due to water rights transfer when the frequency of runoff is more than 50%. Though the compensation fees are decided, the actual situations are more complex including decisions about how and when to compensate and whether the farmer is willing to be compensated rather than planting crops. On the other hand, currently in the process of saved water from canal lining, which contributes the most parts of saved water, governments and enterprises are two critical participants, but the farmers have not been involved directly or even do not comprehend the meaning of water rights transfer^15^, and consequently do not benefit directly from water rights transfer^33^. Besides, the price and period of water rights are guided by governments, thus the transfer in the YRB are regarded as non-market or quasi-market trade^15, 34^. Agriculture is the source of water rights, therefore governments should guarantee the interest of farmers and construct a more free water market to encourage farmers to participate in trading.

In the optimization model, some factors and processes are generalized. The groundwater depth through diversion and transfer is an average depth in a year and ignores the difference of some factors between sub-irrigation areas. In reality, the water demand in the irrigation varies in different seasons and the groundwater depth fluctuates correspondingly^35^. In a sub-irrigation area, the groundwater depth also varies in spatial distribution^36^. The structure of a crop plantation also differs in different sub-irrigation areas^22^. Therefore the temporal and spatial heterogeneous characteristics of the irrigation area should be considered to achieve more precise results. Water rights transfer has successfully started in China and shows a prospective future. Yet at the same time, decision makers should try to minimize negative ecological impacts, and coordinate the relationship of participants to benefit both the buyers and sellers of water rights.

## Methods

### Site description

**Figure 3:**
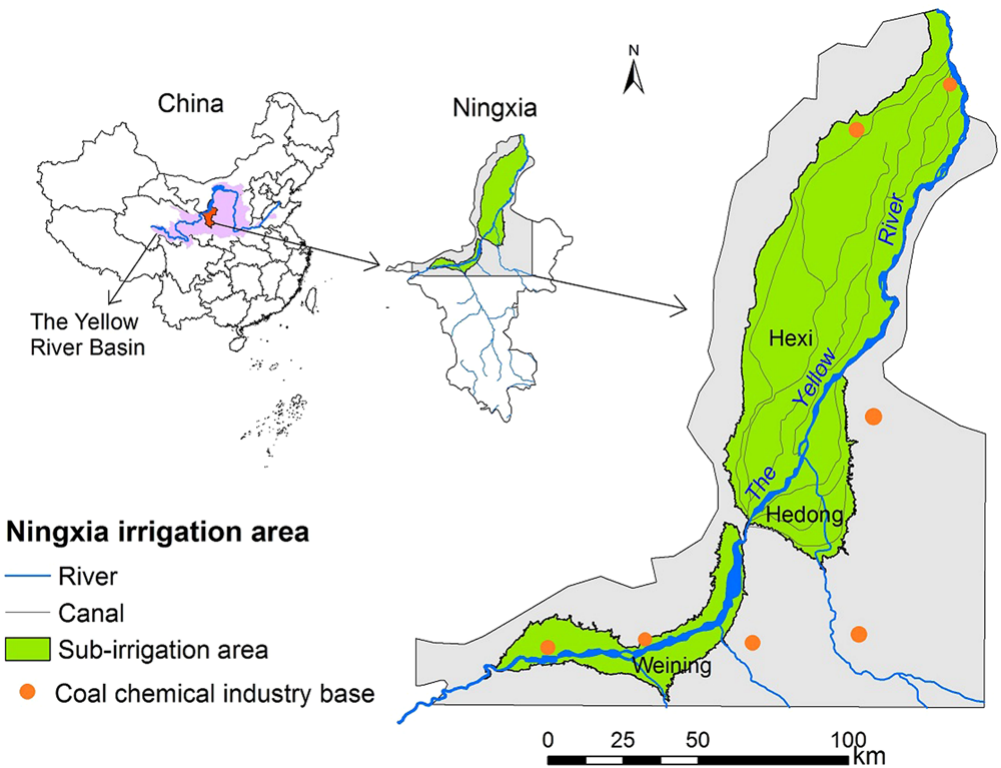
Study site. This figure was generated though ArcGIS 10.2 software provided by Environmental Systems Research Institute (http://www.esri.com).

The Yellow River Irrigation area of Ningxia, or NIA (Ningxia Irrigation Area), lies in the upper stream of the YRB in northwestern China (Fig. 3). It covers an area of 4607 km^2^ with a population of 1.77 million living in countryside^37^. The irrigation area is located in temperate continental climate with an annual average temperature of 8 °C, annual precipitation of 180 mm^38^, and an evapotranspiration capacity of more than 1000 mm. One of the largest coal mines in China is located near the NIA, and an enormous coal chemical industry base is being established. According to the National Medium and Long Term Planning of Coal Chemical Industry Development (Exposure Draft), Ningxia would become one of the eight largest coal chemical bases in China^39^.

The NIA includes three sub-irrigation areas (Hexi, Hedong, and Weining). These areas all contain two types of irrigation areas: in one area, the water must be pumped into the starting canals^37^, and in the other area, the water flows into the irrigation areas directly by gravity^40^. In this study, only the latter one is considered, since it is the major part of NIA, consumes 86% water, and thus has the largest water saving potential. The Yellow River is almost the only source of water for the NIA. In fact, 97.4% water is from the Yellow River, which is 317 km long in Ningxia and has a runoff of 370 × 10^8^ m^3^ when it flows through the NIA.

### Water rights in the site

Not all the water passing through Ningxia belongs to Ningxia. In 1987 the State Council published a regulation and stipulated how much water a province in the YRB can take, which is also called the initial water rights allocation, measured as consumption water. According to this regulation, Ningxia has a right of 40 × 10^8^ m^3^ when the runoff of the Yellow River meets the level of annual average runoff. In other levels, the water rights vary proportionally with the actual runoff. Correspondingly, the water withdrawal from the Yellow River at different frequencies of runoff can be measured (Fig. 2). In 1999~2004, water consumed by Ningxia exceeded the regulation^18^. In 2009, the water rights in Ningxia were allocated legally to the level of county and industry and regulation was issued stating that an enterprise can only obtain water rights from other sectors or other counties by water rights transfer. As a result, no additional water rights can be obtained if extra water is required by an industrial enterprise. In 2004, with the authorization of the Ministry of Water Resources, the governments of Ningxia started to implement the mechanism of water rights transfer, which was the first such strategy in China.

The general practices of water rights transfer in Ningxia is that industrial enterprises invest in agricultural engineering to reduce water usage and then purchase the long-term water rights from agriculture. Based on the regulation that the property rights belong to the nation, the rights for transfer are limited to use and beneficial rights. Physically, the rights are intake rights which means an owner of the rights should use the purchased rights by acquiring water at the intake point and not directly acquiring water from sellers. The capital for investment engineering from industrial enterprises is arranged by local governments and the governments supervise the engineering and coordinate the relationship between agriculture and industry^3^.

By 2015, there are 52 projects implementing or having implemented water rights transfer including 10 coal chemistry projects. These projects will promote the lining rate of irrigation canals from 8% to 19% (Table 1) and transfer 4.94 × 10^8^ m^3^ water to industrial enterprises. According to local planning, there are another 51 coal chemical industry projects that need to transfer water rights. These 51 projects would notably increase the economic level of Ningxia and are the main subjects in our research.

### Data and framework

In this research, the data include water resource data, agriculture data, water rights transfer data, coal chemical industry data, and hydrogeology data. The water resource data includes the groundwater depth, the transferable water, and consumptive water rights allocated under different frequencies of runoff of the Yellow River to each sub-irrigation area. These data were collected from public reports^18, 41^. The agricultural data includes the irrigation area, the structure of crop plantation, output value, and canal system data. These data were collected from the Ningxia Provinsial of Statistics and NBS Survey Office in Ningxia^27^ and the Ningxia Agriculture Department. The water rights transfer data including the current state of lining canals were collected from the Ningxia Water Conservancy. The coal chemical industry data include the water quota and future outputs of coal chemical. All the coal chemical industry projects are focused on the future 51 coal chemical industries that have not been included in the prior water rights transfer but are included in related planning published publicly. Some of these projects are currently being established and some are still being planned. In this study, we assumed that all these 51 projects were established in 2015. The hydrogeology data was obtained mainly from Zhang and Li^42^ and other studies (Table 2).

**Figure 4:**
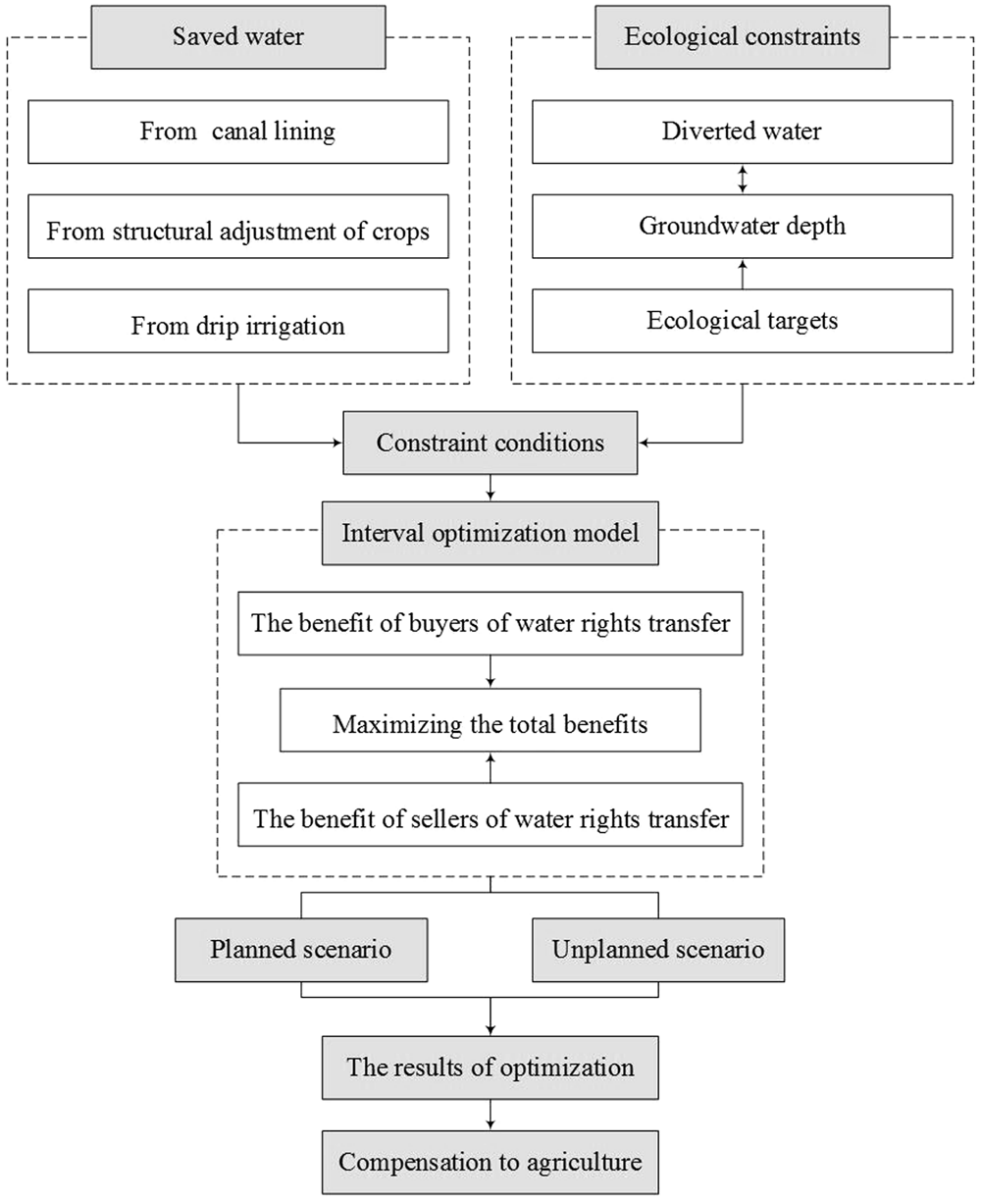
Framework of model.

Figure 4 shows the framework of this model. To establish the model, the saved water from agriculture and the relationship between groundwater depth and diverted water are first determined. Next, these findings are used in an interval optimization model that is established to maximize the total benefits of both the buyers and sellers of water rights transfer subjected to the constraint conditions. Two scenarios, based on whether industrial enterprises are subjected to the water demand of future planned projects, are set to reflect the potential of water rights transfer. Finally, the compensation fees for agriculture will be calculated to analyze the influence of different flow frequencies of the Yellow River.

### The saved water of water rights transfer

Saved water is the base of the Ningxia water rights transfer system. The general practices of the saved water are by using canal lining to decrease the amount of useless leakage, which provides most of the saving potential. Other strategies such as the structural adjustment of crop plantation and using irrigation technology instead of flooding irrigation also can contribute to saving potential. The total water saving *WD* can be expressed as:

1 $$WD=WS+WP+WB$$

where *WS*, *WP*, *WB* represent the saved water from the canal lining, structural adjustment of crop plantation, and using drip irrigation technology, respectively.

### The saved water from canal lining

The canal system has a total length of nearly 40,000 kilometers, which is a complex system (Table 1). It includes five grades of canals: head main canals, main canals, branch canals, lateral canals, and field ditches. Different canals have different length and have a different canal water utilization coefficient. To simplify the calculation, we use an equal canal system method to generalize the system. The core idea of this method is to equate the canal water utilization coefficient of each grade canals to the head main canals. By measuring the differential head water into the irrigation area, the amount of saved water can be calculated. The detailed justification of the equal canal system method is described in the supplementary information.

All the volumes or quantities of water rights for transfer are measured as the consumption water which means the water consumed by users. Thus there are typically two different terms in the NIA’s water rights transfer system, water saving potential (∑*WS*_*i*_ or *WD*) and transferable water rights (*WT*). The former one refers to the water that is saved as measured at the intake point, the latter one refers to the consumed water based on the water saving potential. There is a conversion coefficient *λ* correlating these factors.

2 $$WT=\lambda WD.$$

The water saved from canal lining *WS* is:

3 $$WS=\sum _{i=1}^{m}W{S}_{i}=\sum _{i=1}^{m}W{Y}_{i}(1-{\eta }_{i}^{\text{'}}/{\eta }_{i}(X))=\sum _{i=1}^{m}W{Y}_{i}(1-{\eta }_{i}^{\text{'}}/\prod _{j=0}^{n}{\eta }_{i,j}({x}_{ij})).$$

where *WS*_*i*_, *WY*_*i*_ represent the amount of water saved and the diverted water of the *i*_*th*_ irrigation, 10^8^ m^3^; *η*_*i*_^'^,*η*_*i*_(*X*) is the initial and expected canal water utilization coefficient in *i*_*th*_ irrigation in the equal canal method; *η*_*i*,*j*_(*x*_*ij*_) is the *j*_*th*_ grade canal water utilization coefficient in the *i*_*th*_ irrigation in the equal canal method; ${x}_{ij}^{\pm }$ (*i* = 1, 2, …, *m*; *j* = 1, 2, …, *n*) is the lining rate of the *j*_*th*_ grade canal in the *i*_*th*_ sub-irrigation.

Ideally all the canals would all be lined to reach the maximum canal water utilization coefficient and water-saving potential. In reality, the canal water utilization coefficient will be smaller if the lining rate of the canals are lower. The canal water utilization coefficient would increase by 0.02 when the lining rate of the canals (except the field ditches) increases by 10%^43^. As for the filled ditches, the canal water utilization coefficient would increase by 0.05^44^. Therefore, equation (3) can be written as (the detail deduced processes can be referred to supplementary information):

4 $$WS=\sum _{i=1}^{m}W{Y}_{i}(1-{\eta }_{i}^{\text{'}}/({\eta }_{i}\prod _{j=1}^{4}(0.2{x}_{ij}+0.8)(0.5{x}_{i5}+0.5))).$$

where *η*_*i*_ is the canal water utilization coefficient when all the canals are lined. *x*_*ij*_(*j* = 1, 2, 3, 4, 5) represents the lining rate of five grades canals (head main canals, main canals, branch canals, lateral canals, and filled ditches) in the *i*_*th*_ irrigation area.

### The saved water from structural adjustment of crop plantation

Different crops consume different amounts of water. By optimizing the structure of crop plantation, water can be saved. The saved water from structural adjustment comes from the differential of water before and after water adjustment. And the water of crops needs in filed based on their area and irrigation quota.

5 $$WP=\sum _{s=1}^{S}{m}_{s}A{x}_{s}^{\text{'}}-\sum _{s=1}^{S}{m}_{s}A{x}_{s}=\sum _{s=1}^{S}{m}_{s}A({x}_{s}^{\text{'}}-{x}_{s}).$$

where *x*_*s*_^'^, *x*_*s*_(*s* = 1, 2, …, *S*) are the current and optimized the proportion of planting *s*_*th*_ crop in the whole irrigation area respectively; *m*_*s*_ is irrigation quota of the *s*_*th*_ crop m^3^; *A* is the area of the total irrigation district, mu (a Chinese unit of area, a mu equals 667 m^2^).

### The saved water from using drip irrigation technology

In the NIA, nearly all the fields are irrigated by flooding. This traditional irrigation method results in significant water waste. Drip irrigation technology can effectively reduce the waste for some crops such as corn and wheat. This technology is highly advocated in Ningxia, which requires the investment of industrial enterprises but is a promising technology. The amount of saved water from this technology can be shown as:

6 $$WB=\sum _{t=s+1}^{T}{m}_{t}A{x}_{t}-\sum _{t=s+1}^{T}{m}_{a,t}A{x}_{t}=\sum _{t=s+1}^{T}({m}_{t}-{m}_{a,t})A{x}_{t}.$$

where to distinguish the subscript from the number of crops of structural adjustment, the initial subscript of crops requiring drip irrigation is set as *s* + *1*; *x*_*t*_ are the proportion of using dripping technology for *t*_*th*_ crop in the whole irrigation area; *m*_*t*_, *m*_*a*,*t*_ is the current irrigation quota of the *t*_*th*_ crop, and the irrigation quota after using dripping technology, m^3^.

Therefore the transferable water *WT* could be measured as:

7 $$\begin{array}{rcl}WT & = & \lambda WD=\lambda (WS+WP+WB)\\  & = & \lambda (\sum _{i=1}^{m}W{Y}_{i}(1-{\eta }_{i}^{\text{'}}/{\eta }_{i}\prod _{j=1}^{4}(0.2{x}_{ij}+0.8)(0.5{x}_{i5}+0.5))\\  &  & +\,\sum _{s=1}^{S}{m}_{s}A({x}_{s}^{\text{'}}-{x}_{s})+\sum _{t=s+1}^{T}({m}_{t}-{m}_{a,t})A{x}_{t})\end{array}$$

### The relationship between surface water diversion and groundwater depth

Water rights transfer implemented in the NIA is based on saved agricultural water. Once the diverted surface water (∑*WY*_*i*_) flowing into the irrigation area decreases, there will be significant terrestrial ecological impacts in the NIA such as desertification, salinization, and a reduction in crop outputs. All these ecological factors are closely related to groundwater levels. In other words, surface ecological condition can be assessed by ecological groundwater levels.

The groundwater balance in irrigation area can be represented as recharge equals discharge as:

8 $${W}_{pr}+{W}_{cr}+{W}_{fr}+{W}_{wr}+{W}_{lr}={W}_{ed}+{W}_{ld}+{\rm{\Delta }}{W}_{g}+{W}_{wd}.$$

where *W*_*pr*_, *W*_*cr*_, *W*_*fr*_, *W*_*wr*_, *W*_*lr*_ are the recharges of precipitation, seepage of canals, seepage of fields, wells, and lateral recharge respectively; *W*_*ed*_, *W*_*ld*_, Δ*W*_*g*_, *W*_*wd*_ are the phreatic water evapotranspiration, the lateral discharge, the variance of groundwater caused by water rights transfer, and the exploitation by wells, respectively.

The NIA has plain topography and the lateral water exchange (*W*_*lr*_ = *W*_*ld*_) is balanced. The volume from the exploitation by wells is much less than the volume of others and thus *W*_*wr*_ and *W*_*wd*_ can be ignored. Therefore the equation can be written as:

9 $${W}_{pr}+{W}_{cr}+{W}_{fr}={W}_{ed}+{\rm{\Delta }}{W}_{g}$$

and

10 $${W}_{pr}=\alpha PF$$

11 $${W}_{cr}=mW=r(1-\eta )W$$

12 $${W}_{fr}=\beta {W}_{f}=k(1-{\eta }_{f}){W}_{f}=k(1-{\eta }_{f})\eta W$$

13 $${W}_{ed}=c{E}_{0}F$$

14 $${\rm{\Delta }}{W}_{g}=\mu (h-{h}_{0})F$$

where *α* represents the recharge coefficient of precipitation, *P* is the precipitation (m), *F* is the area of irrigation (km^2^), *m* is the recharge coefficient of seepages of canals, *W* is the volume of diverted water in the starting canals from the Yellow River (10^8^m^3^), *W*_*f*_ is the volume of irrigation into the field (10^8^m^3^), *r* is the correction coefficient of the recharge of the seepages of canals, *η* is the coefficient of the utilization of canal water, *η*_*f*_ is the coefficient of the utilization of field water, *β* is the coefficient of irrigation recharge in filed, *k* is the correction coefficient of groundwater recharge in the field, *c* is the coefficient of phreatic water evapotranspiration, *E*_0_ is the evapotranspiration capacity (m), *μ* is the coefficient of recharge, and *h* and *h*_*0*_ represent the groundwater depth after and before water rights transfer (m).

Therefore, the equation can be expressed as:

15 $$0.01\alpha PF+r(1-\eta )W+k(1-{\eta }_{f})\eta W=0.01c{E}_{0}F+0.01\mu (h-{h}_{0})F$$

This equation can be rewritten as the relationship between groundwater depth and surface water diversion:

16 $$W=\frac{0.01F[c{E}_{0}+\mu (h-{h}_{0})-\alpha P]}{r(1-\eta )+k(1-{\eta }_{f})\eta }$$

in this equation, *h*, *η* is variable, *E*_0_, *P*, *h*_0_, *η*_*f*_ is constant, other hydrogeological parameters are obtained from related hydrogeological references experiments, and the values of *c*, *μ*, *α*, *k* are related to ground depth or the variable *h*. Therefore it is critical to determine the groundwater depths controlling the ecology in the NIA. To do this, the relative studies were reviewed, and six targets were chosen as ecological goals in the NIA (Table 2).

Before determining the values coordinated with the ecological targets, it is essential to calculate the values of *c*, *μ*, *α*, *k* for the corresponding groundwater depths. The calculations are based on experiments done in the NIA^45, 46^. And then the data is fitted with fitting curves, which allows the values of these parameters in any groundwater depth to be calculated (the fitting figures and their equations are shown in the supplementary information).

### Interval optimization model

The decision variable *X* ^±^ includes three types of variables: ${x}_{ij}^{\pm }$ (*i* = 1, 2, …, *m*; *j* = 1, 2, …, *n*) describing the lining rate of the *j*_*th*_ grade canal in the *i*_*th*_ sub-irrigation; ${x}_{s}^{\pm }$ (*s* = 1, 2, …, *S*) are the proportion of planting *s*_*th*_ crop in the whole irrigation area; ${x}_{t}^{\pm }$ (*t* = 1, 2, …, *T*) are the proportion of using dripping technology for *t*_*th*_ crop in the whole irrigation area.

The objective function aims to maximize the benefits of water rights transfer system *F(x)*. This includes the benefits of agriculture ${F}_{1}^{\pm }(X)$ and the coal chemical industry ${F}_{2}^{\pm }(X)$.

17 $${\rm{\max }}\,{F}^{\pm }(X)={F}_{1}^{\pm }(X)+{F}_{2}^{\pm }(X)$$

### The benefits of agriculture

There are three components of benefits from agriculture ${F}_{1}^{\pm }(X)$: the income of structural adjustment ${f}_{c}^{\pm }(X)$, the income of selling the saved water from structural adjustment of crop plantation $\,{f}_{p}^{\pm }(X)$, and the benefit of increased production as a result of drip irrigation ${f}_{r}^{\pm }(X)$.

18 $${F}_{1}^{\pm }(X)={f}_{c}^{\pm }(X)+{f}_{p}^{\pm }(X)+{f}_{r}^{\pm }(X)$$

The income of structural adjustment of crop plantation:

19 $${f}_{c}^{\pm }(X)=\sum _{s=1}^{S}{r}_{s}^{\pm }A{x}_{s}^{\pm }$$

where *s* means there are *s* kinds of crops, *r*_*s*_ is the benefit of the *s*_*th*_ crop per mu, 10^8^ yuan.

The income of selling the saved water from structural adjustment of crop plantation:

20 $${f}_{p}^{\pm }(X)={\lambda }^{\pm }{R}_{w}W{P}^{\pm }$$

where *λ* is the conversion coefficient of saved water from diversion to consumption, *R*_*w*_ is the price of agricultural water, yuan/m^3^, *WP* is the saved water from structural adjustment of crop plantation.

The benefit of increased production as a result of drip irrigation:

21 $${f}_{r}^{\pm }(X)=\sum _{t=s+1}^{T}{\alpha }^{\pm }{r}_{t}^{\pm }A{x}_{t}^{\pm }$$

where *t* means there are (*t-s*) kinds of crops using drip irrigation technology, *α* is the increasing percent of using drip irrigation compared with not using of irrigation for crops, *r*_*t*_ is the benefit of the *t*_*th*_ crop per mu.

### The benefits of coal chemical industry

The benefits of coal chemical industry ${F}_{2}^{\pm }(X)$ is also composed of three parts: the benefits of selling the production of coal chemistry ${F}_{d}^{\pm }(X)$, the expenditure of investing in the water saving engineering ${F}_{w}^{\pm }(X)$, and the expenditure of buying the saved water from the structural adjustment of crop plantation ${F}_{p}^{\pm }(X)$.

22 $${F}_{2}^{\pm }(X)={f}_{d}^{\pm }(X)-{f}_{w}^{\pm }(X)-{f}_{p}^{\pm }(X)$$

The benefits of selling the production of coal chemistry:

23 $${f}_{d}^{\pm }(X)=\frac{W{T}^{\pm }}{{m}_{c}^{\pm }}{R}_{c}^{\pm }\xi $$

where *WT* denotes the transferable water saved, 10^8^m^3^; *m*_*c*_ is the average water quota of coal chemical industry, 10^8^m^3^/10^4^t; *R*_*c*_ is the value per output of coal chemistry, 10^8^ yuan/10^4^t; *ξ* is the average rate of profit of coal chemical product (not including the expenditure of water saving engineering).

The expenditure of investing in water saving engineering, including canal lining and implementing drip irrigation technology for agriculture:

24 $${f}_{w}^{\pm }(X)=\sum _{i=1}^{m}\sum _{j=1}^{n}{R}_{e,j}^{\pm }{L}_{ij}({x}_{ij}^{\pm }-{x}_{ij}^{\text{'}})+\sum _{t=1}^{T}{R}_{f}^{\pm }A{x}_{t}^{\pm }$$

*R*_*e*,*j*_ is the investment of lining the *j*_*th*_ grade canal per km, 10^8^ yuan/km; *L*_*ij*_ is the length of the *j*_*th*_ grade canal in the *i*_*th*_ irrigation, km; *x*′_*ij*_ is the initial lining rate for *j*_*th*_ grade canal in the *i*_*th*_ irrigation; and *R*_*f*_ is the investment of drip technology per unit area, 10^8^ yuan/10^4^mu.

Therefore, the objective function is summarized as:

25 $${F}^{\pm }(X)=\sum _{s=1}^{S}{r}_{s}^{\pm }A{x}_{s}^{\pm }+\sum _{t=s+1}^{T}{\alpha }^{\pm }{r}_{t}^{\pm }A{x}_{t}^{\pm }+\frac{W{T}^{\pm }}{{m}_{c}^{\pm }}{R}_{c}^{\pm }\xi -\sum _{i=1}^{m}\sum _{j=1}^{n}{R}_{e,j}^{\pm }{L}_{ij}({x}_{ij}^{\pm }-{x}_{ij}^{\text{'}})-\sum _{t=s+1}^{T}{R}_{f}^{\pm }A{x}_{t}^{\pm }$$

where the transferable water rights *WT* can be expressed according to equation (7):

26 $$W{T}^{\pm }={\lambda }^{\pm }(\begin{array}{c}\sum _{i=1}^{m}W{Y}_{i}(1-{\eta }_{i}^{\text{'}}/{\eta }_{i}\prod _{j=1}^{5}(0.2{x}_{ij}^{\pm }+0.8)(0.5{x}_{i5}^{\pm }+0.5))+\sum _{s=1}^{S}{m}_{s}^{\pm }A({x}_{s}^{\text{'}}-{x}_{s}^{\pm })\\ +\,\sum _{t=s+1}^{T}({m}_{t}^{\pm }-{m}_{a,t}^{\pm })A{x}_{t}^{\pm }\end{array})$$

### Constraint conditions

The constraint conditions of water transfer system in the NIA include natural constraints, water-saving measures limitations, economic constraints, and nonnegative constraints.

27 $$0\le {x}_{t}^{\pm } < {x}_{s}^{\pm }\le 1(s=1,\mathrm{..},S,s=t+1,\mathrm{...},T)$$

28 $${x}_{ij}^{\text{'}}\le {x}_{ij}^{\pm }\le 1$$

29 $${\sum }_{s=1}^{S}{x}_{s}^{\pm }=1$$

30 $$W{T}^{\pm }\le W{T}_{0}$$

31 $$W{T}^{\pm }\le W{T}_{e}^{\pm }$$

32 $$W{T}^{\pm }\le W{T}_{c}^{\pm }$$

33 $$\sum _{s=1}^{S}{m}_{i}A({x}_{s}^{\text{'}}-{x}_{s}^{\pm })\ge 0$$

34 $$\sum _{s=1}^{S}{r}_{s}^{\pm }A{x}_{s}^{\pm }\ge \sum _{s=1}^{S}{r}_{s}^{\pm }A{{x}_{s}}^{^{\prime} }$$

35 $$\sum _{s=1}^{S}{m}_{s}^{\pm }A({{x}_{s}}^{^{\prime} }-{x}_{s}^{\pm })\le W{P}_{0}$$

36 $$\sum _{t=s+1}^{T}({m}_{t}^{\pm }-{m}_{a,t}^{\pm })A{x}_{t}^{\pm }\le W{B}_{0}$$

37 $$\sum _{i=1}^{m}W{Y}_{i}(1-{\eta }_{i}^{\text{'}}/{\eta }_{i}\prod _{j=1}^{4}(0.2{x}_{ij}^{\pm }+0.8)(0.5{x}_{i5}^{\pm }+0.5))\le W{S}_{0}$$

38 $$\sum _{i=1}^{m}\sum _{j=1}^{n}{R}_{e,j}^{\pm }{L}_{ij}({x}_{ij}^{\pm }-{{x}_{ij}}^{^{\prime} })+\sum _{t=s+1}^{T}{R}_{f}^{\pm }A{x}_{t}^{\pm }+{\lambda }^{\pm }{R}_{w}\sum _{s=1}^{S}{m}_{i}^{\pm }A({{x}_{t}}^{^{\prime} }-{x}_{t}^{\pm })\le \frac{W{T}^{\pm }}{{m}_{c}^{\pm }}{R}_{c}^{\pm }\xi $$

The inequation (27) means the percent of area of a crop developing dripping irrigation can not exceed their corresponding planted percent of area; inequation (28) means the lining rate of any grade canal should not be less than the current level; inequation (29) means the planted proportion of area of all the crops remain constant; inequation (30) means the transferable water *WT* cannot surpass the volumes currently allocated to agriculture in the Ningxia initial water rights allocation *WT*_*0*_; inequation (31) means *WT* cannot surpass the ecological constraint $W{T}_{e}^{\pm };$ inequation (32) means the transferable water is less than the theoretical water demand of industrial enterprises; inequation (33) means the water demand of crop should not exceed current volumes; inequation (34) means the benefit after structural adjustment of crop plantation should increase; inequation (35), (36) and (37) means the saved water from the canal lining *WS*, structural adjustment of crop plantation *WP*, and using drip irrigation technology *WB* cannot exceed their potentials *WS*_*0*_, *WP*_*0*_, *WB*_*0*_; inequation (38) means the investment in lining, dripping and the expenditure of purchasing farmers’ saved water cannot exceed the average rate of profit *ξ*.

### Values of parameters and modeling solution

The values of main interval parameters of NIA water rights transfer system are shown in Table 4. The interval function can be calculated to separately obtain the upper bound *F* ^+^(*X*) and lower bound *F* ^−^(*X*). The detailed solution can be referred to supplementary information. Then, the result of the decision variables is $X={(({x}_{s}^{-},{x}_{s}^{+}),({x}_{t}^{-},{x}_{t}^{+}),({x}_{ij}^{-},{x}_{ij}^{+}))}^{T}$.

**Table 4:** The values of interval parameters.

Parameter	Symbol	Unit	Value
Benefit of the *s*_*th*_ crop per mu	*r*_*s*_^±^, *r*_*t*_^±^(*t* = *s*+1)	10^8^ yuan	*r*_1_^±^ = [−0.0242, 0.0132] *r*_2_^±^ = [0.0652, 0.1007] *r*_3_^±^ = [0.929, 0.1116]
Increasing percent of using drip irrigation	*α* ^±^	—	[14%, 17.2%]
Average water quota of coal chemical industry	*m*_*c*_^±^	10^8^ m^3^/10^4^t	[5.8, 7.5] × 10^−4^
Value per output of coal chemistry	*R*_*c*_^±^	10^8^ yuan/10^4^t	[0.665, 1.083]
Investment of lining the *j*_*th*_ grade canal per km	*R*_*e*,*j*_^±^	10^8^ yuan/km	*R*_*e*,1_^±^ = [221, 281]*R*_*e*,2_^±^ = [60, 138]*R*_*e*,3_^±^ = [19, 42]*R*_*e*,4_^±^ = [3, 15]*R*_*e*,5_^±^ = [4, 10]
Investment of dripping technology per unit area	*R*_*f*_^±^	10^8^ yuan/10^4^mu	[0.05, 0.13]
Net irrigation water quota of the *s*_*th*_ crop	*m*_*s*_^±^, *m*_*t*_^±^	m^3^	*m*_1_^±^ = [290, 310] *m*_2_^±^ = [270, 290] *m*_3_^±^ = [790, 830]
Irrigation water quota of the *t*_*th*_ crop after using dripping technology	*m*_*a*,*t*_^±^	m^3^	*m*_*a*,1_^±^ = [218, 233] *m*_*a*,2_^±^ = 140
Conversion coefficient of saved water from diversion to consumption	*λ* ^±^	—	[0.33, 0.36]
Total water demand of coal chemical enterprises	*W*_*c*_^±^	10^8^ m^3^	[4.24, 5.44]

### The compensation of water rights transfer under different water frequencies

The quantity of water rights transfer is based on annual average runoff. In addition, the guarantee of industrial water is higher than agricultural water. Therefore, when the runoff of the Yellow River *W*_*p*_ is less than the average level *W*_*n*_, agriculture would suffer the risk of loss. Thus, it will be necessary to compensate the loss from industry to agriculture.

The compensation fee *C* is the difference between the loss of crops and the water charge that should be paid:

39 $$C=\sum _{i=1}^{m}{\varepsilon }_{i}{x}_{i}{R}_{i}A$$

40 $${\varepsilon }_{i}=\frac{{m}_{i}A{x}_{i}}{\sum _{i=1}^{3}{m}_{i}A{x}_{i}}$$

41 $$A={\rm{\Delta }}WT/{m}_{sy}$$

where *R*_*i*_ denotes the difference of the benefit of per area (mu) using irrigation or not, *yuan*; *m*_*sy*_ is the synthetical net irrigation water quota, *m*_*i*_ is the net irrigation water quota of the *i*_*th*_ crop m^3^/mu; *ε*_*i*_ is the coefficient of water sharing ratio of the *i*_*th*_ crop; *A* is the total irrigation area in the NIA, 10^5^ mu; The shortage water rights of agriculture Δ*WT* due to water rights transfer is:

42 $${\rm{\Delta }}WT=WT-\frac{{W}_{p}}{{W}_{n}}WT$$

Therefore the compensation fee can be expressed as:

43 $$C=(\sum _{i=1}^{m}\frac{{m}_{i}{x}_{i}^{2}{R}_{i}}{\sum _{i=1}^{m}{m}_{i}{x}_{i}})(1-\frac{{W}_{p}}{{W}_{n}})\frac{WT}{{m}_{sy}}$$

## Electronic supplementary material

Supplementary information
