# Supplementary material for: Interval Optimization Model Considering Terrestrial Ecological Impacts for Water Rights Transfer from Agriculture to Industry in Ningxia, China
Source: Sci Rep. 2017 Jun 14;7:3465. doi: 10.1038/s41598-017-02734-9 (PMC5471265; doi:10.1038/s41598-017-02734-9)
Supplement: Supplementary file 1 — Supplementary information [file 41598_2017_2734_MOESM1_ESM.pdf]

Interval Optimization Model Considering Terrestrial Ecological Impacts for Water Rights Transfer from Agriculture to Industry in Ningxia, China

Lian Sun[1,3], Chunhui Li[1,*], Yanpeng Cai[2,3] & Xuan Wang[2,3]

[1]Key Laboratory for Water and Sediment Sciences of Ministry of Education, School of Environment, Beijing Normal University, Beijing 100875, China; [2]Beijing Engineering Research Center for Watershed Environmental Restoration & Integrated Ecological Regulation, School of Environment, Beijing Normal University, Beijing 100875, China; [3]State Key Laboratory of Water Environment Simulation, Beijing Normal University, Beijing 100875, China. Correspondence and requests for materials should be addressed to C.L. (email: chunhuili@bnu.edu.cn)

The supplementary information includes four sections:
Equal canal method for calculating water-saving potential
Formulation of equation (4)
Determination of hydrogeological parameters
Modeling solution

Equal canal method for calculating water-saving potential

The saved water of canal system is based on the equal canal method[s1] that requires relatively less data. There are three main steps of this method.

The first step is to generalize different grades of canals in an irrigation region into equal canals of equal length and equal runoff.

Equal length $l_i^{'}$ can be written:

$$l_i^{'} = \frac{\sum l_i}{n_i} \tag{s1}$$

where l_i is the length of the i_{th} grade canals.km; n_i is the number of the i_{th} canals.

Equal runoff $Q_i^{'}$ can be written as:

$$Q_i^{'} = \frac{W_Y \times 10^4}{8.64 T_I n_i} \tag{s2}$$

where W_Y is the water in the head of the canal system, 10^8 m^3; T_I designates the operation time of the i_{th} grade canals.

The second step is to analyze the canal water utilization coefficient of the equal canal according to the experiment of the loss of transferring water.

$$\frac{S_1}{S_2}=\frac{A_1Q_1^{1-m_1}}{A_2Q_2^{1-m_2}} \quad \text{(s3)}$$

where S_1 and S_2 represent the loss water of the equal canal and a typical experimental region, respectively, $m^3/(s.km)$; Q_1, Q_2 are the runoff of the equal canal and the typical experimental region, m^3/s; A_1, A_2 are the soil porous coefficients of the equal canal and the typical experimental region; and m_1, m_2 are the soil porous indices of the equal canal and the typical experimental region.

Then the water utilization coefficient of a unit length of the equal canal η_{iu} can be written as:

$$\eta_{iu}=l_i^{'}\sqrt{\frac{Q_i^{'}-l_i^{'}S_{f1i}}{Q_i^{'}}} \quad \text{(s4)}$$

where S_{f1i} represents the water loss of the unit length of the i_{th} grade canal, $m^3/(s.km)$. S_{f1i} relates to the experience equation based on the experiment performed by the Ningxia Water Conservancy:

Name of canal	Water loss with no canal lining	Water loss with canal lining
Yuejin Canal	$S=0.050Q^{0.4}$	$S=0.014Q^{0.4}$
Donggan Canal	$S=0.050Q^{0.4}$	$S=0.014Q^{0.4}$
Xigan Canal	$S=0.045Q^{0.4}$	$S=0.014Q^{0.4}$
Tanglai Canal	$S=0.035Q^{0.4}$	$S=0.014Q^{0.4}$
Hanyan Canal	$S=0.040Q^{0.4}$	$S=0.014Q^{0.4}$
Huinong Canal	$S=0.030Q^{0.4}$	$S=0.014Q^{0.4}$

Table S1. The experience equation of the water transfer experiment ($m^3 \cdot s^{-1} \cdot km^{-1}$ **).** Data are from Ningxia Water Conservancy[s2].

Formulation of equation (4)

Equation 3 in the section of method is:

$$WS=\sum_{i=1}^{m}WS_i=\sum_{i=1}^{m}WY_i(1-\eta_i^{'}/\eta_i(X))=\sum_{i=1}^{m}WY_i(1-\eta_i^{'}/\prod_{j=0}^{n}\eta_{i,j}(x_{ij})) \quad (3)$$

Since the canal water utilization coefficient would increase by 0.02 (for 1st to 4th grade canals) and by 0.05 (for 5th grade canals) when the lining rate of the canals increases by 10% 30, equation 3 will be adjusted. Take the first canal for example. The first grade canal water utilization coefficient is $\eta_{i,1}(x_{i,1})$ when these grade canals in i_{th}

irrigation district are all lined (e.g. the lining rate of the first grade canal is $x_{i,1}=1$). When $x_{i,1}=0.9$, $x_{i,1}=0.8$, the grade canal water utilization coefficient is $(1-0.02)\eta_{i,1}(x_{i,1})$, $(1-0.02\times 2)\eta_{i,1}(x_{i,1})$ respectively. Therefore for any given lining rate $x_{i,1}$, its corresponding water utilization coefficient is $(0.2x_{i,1}+0.8)\eta_{i,1}$. Thus, for any grade canal, corresponding water utilization coefficient is $(0.2x_{i,j}+0.8)\eta_{i,j}\ (j=1,2,3,4)$. Similarly, for the filled ditches (e.g. 5th grade canals) the canal water utilization coefficient is $(0.5x_{i,5}+0.5)\eta_{i,5}$. Besides, the whole water utilization coefficient η_i is:

$$\eta_i=\prod_{j=0}^{5}\eta_{i,j} \tag{s5}$$

Thus, for any given lining rate of i_{th} irrigation district, the actual water utilization efficient is:

$$\prod_{j=0}^{5}\eta_{i,j}(x_{ij})=\eta_i\prod_{j=1}^{4}(0.2x_{ij}+0.8)(0.5x_{i5}+0.5)) \tag{s6}$$

Therefore, equation (3) can be written as:

$$WS=\sum_{i=1}^{m}WY_i(1-\eta_i^{'}/(\eta_i\prod_{j=1}^{4}(0.2x_{ij}+0.8)(0.5x_{i5}+0.5))) \tag{4}$$

Determination of hydrogeological parameters

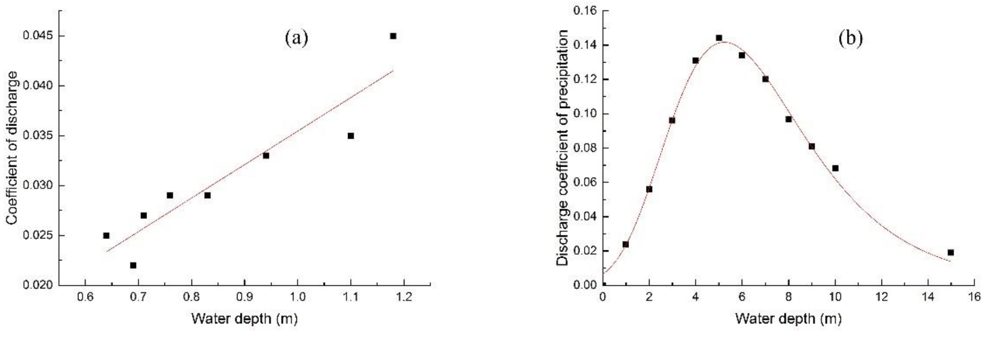

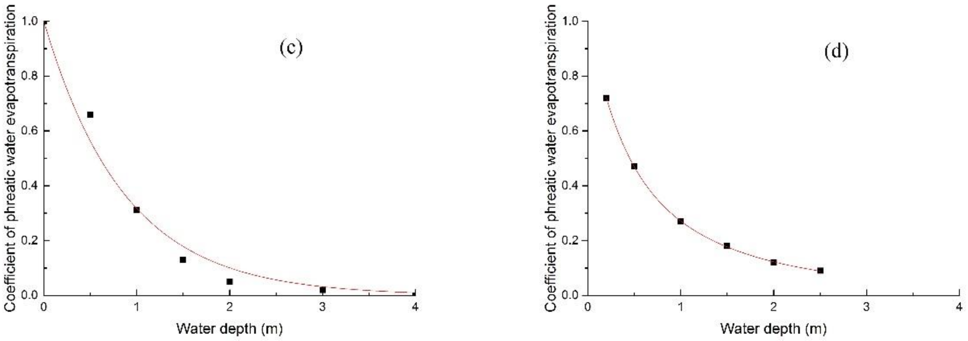

Figure S1. Fitting curves of hydrogeological parameters based on field experiments by Ren et al.[s3] and Gao et al.[s4]

(a): Coefficient of recharge in the NIA: $\mu(h) = 0.00188 + 0.03357h$, $R^2 = 0.992$;

(b): Recharge coefficient of precipitation in the NIA:

$\alpha(h) = 0.00108 + 0.14062\exp(-\exp(-z(h)) - z(h) + 1)$, $R^2 = 0.857$

where $z(h) = (h - 5.22947)/2.90869$;

(c): Coefficient of phreatic water evapotranspiration in Hexi sub-irrigation district:

$c(h) = e^{-1.147h}$, $R^2 = 0.983$;

(d): Coefficient of phreatic water evapotranspiration in Hedong sub-irrigation district: $c(h) = 1.01168 - 0.47454(1 - e^{-h/0.336}) - 0.51189(1 - e^{-h/1.19744})$, $R^2 = 0.999$.

Modeling solution

The water utilization coefficient η_i is expressed as:

$$\eta_i = \eta_{iu}^{l_i'} \tag{s7}$$

Water utilization coefficient of the equal canal system η_α

$$\eta_\alpha = \prod_{i=1}^{n} \eta_i \tag{s8}$$

(1) The third step is to calculate the saved water of the canal system W_S:

$$W_S = \sum_{i=1}^{m} WY_i(1 - \eta_b / \eta_a) \tag{s9}$$

where WY_i is the diverted water of the i_{th} irrigation; η_a, η_b represents the water utilization coefficient of the equal canal system before and after canal lining.

The upper bound:

$$\max F^+(X) = \sum_{s=1}^{S} r_s^+ Ax_s + \sum_{t=1}^{T} \alpha^+ r_t^+ Ax_t + \frac{WT^+}{m_c^-} R_c^+ \xi - \sum_{i=1}^{m}\sum_{j=1}^{n} R_{e,j}^- L_{ij}(x_{ij} - x_{ij}^{'}) - \sum_{t=1}^{T} R_f^- Ax_t$$

$$WT^+ = \lambda^+ \left(\sum_{i=1}^{m} WY_i (1 - \eta_i^{'} / \eta_i \prod_{j=1}^{4} (0.2x_{ij} + 0.8)(0.5x_{i5} + 0.5)) + \sum_{s=1}^{S} m_s^+ A(x_s^{'} - x_s) + \sum_{t=1}^{T} (m_t^+ - m_{a,t}^-) Ax_t \right)$$

$$0 \le x_t < x_s \le 1 \, (s = 1,..,S, s = t+1,...,T) \quad \text{(s10)}$$

$$x_{ij}^{'} \le x_{ij} \le 1 \quad \text{(s11)}$$

$$\sum_{s=1}^{S} x_s = 1 \quad \text{(s12)}$$

$$WT^+ \le WT_0 \quad \text{(s13)}$$

$$WT^+ \le WT_e^+ \quad \text{(s14)}$$

$$WT^+ \le WT_c^+ \quad \text{(s15)}$$

$$\sum_{s=1}^{S} m_i A(x_s^{'} - x_s) \ge 0 \quad \text{(s16)}$$

$$\sum_{s=1}^{S} r_s^+ Ax_s \ge \sum_{s=1}^{S} r_i^+ Ax_s' \quad \text{(s17)}$$

$$\sum_{s=1}^{S} m_s^+ A(x_s' - x_s) \le WP_0 \quad \text{(s18)}$$

$$\sum_{t=s+1}^{T} (m_t^+ - m_{a,t}^-) Ax_t \le WB_0 \quad \text{(s19)}$$

$$\sum_{i=1}^{m} WY_i (1 - \eta_i^{'} / \eta_i \prod_{j=1}^{4} (0.2x_{ij} + 0.8)(0.5x_{i5} + 0.5)) \le WS_0 \quad \text{(s20)}$$

$$\sum_{i=1}^{m}\sum_{j=1}^{n} R_{e,j}^- L_{ij}(x_{ij} - x_{ij}') + \sum_{t=1}^{T} R_f^- Ax_t + \lambda^+ R_w \sum_{s=1}^{S} m_i^+ A(x_t' - x_t) \le \frac{WT^+}{m_c^-} R_c^+ \xi \quad \text{(s21)}$$

The lower bound:

$$\max F^-(X) = \sum_{s=1}^{S} r_s^- Ax_s + \sum_{t=1}^{T} \alpha^- r_t^- Ax_t + \frac{WT^-}{m_c^+} R_c^- \xi - \sum_{i=1}^{m}\sum_{j=1}^{n} R_{e,j}^+ L_{ij}(x_{ij} - x_{ij}^{'}) - \sum_{t=s+1}^{T} R_f^+ Ax_t$$

$$WT^- = \lambda^- \left(\sum_{i=1}^{m} WY_i(1-\eta_i^{'}/\eta_i \prod_{j=1}^{4}(0.2x_{ij}+0.8)(0.5x_{i5}+0.5)) + \sum_{s=1}^{S} m_s^- A(x_s^{'}-x_s) + \sum_{t=s+1}^{T}(m_t^- - m_{a,t}^+)Ax_t \right)$$

$$0 \le x_t < x_s \le 1\,(s=1,..,S, s=t+1,...,T) \quad \text{(s22)}$$

$$x_{ij}^{'} \le x_{ij} \le 1 \quad \text{(s23)}$$

$$\sum\nolimits_{s=1}^{S} x_s = 1 \quad \text{(s24)}$$

$$WT^- \le WT_0 \quad \text{(s25)}$$

$$WT^- \le WT_e^- \quad \text{(s26)}$$

$$WT^- \le WT_c^- \quad \text{(s27)}$$

$$\sum_{s=1}^{S} m_i A(x_s^{'} - x_s) \ge 0 \quad \text{(s28)}$$

$$\sum_{s=1}^{S} r_s^- Ax_s \ge \sum_{s=1}^{S} r_s^- Ax_s' \quad \text{(s29)}$$

$$\sum_{s=1}^{S} m_s^- A(x_s' - x_s) \le WP_0 \quad \text{(s30)}$$

$$\sum_{t=1}^{T}(m_t^- - m_{a,t}^+)Ax_t \le WB_0 \quad \text{(s31)}$$

$$\sum_{i=1}^{m} WY_i(1-\eta_i^{'}/\eta_i \prod_{j=1}^{4}(0.2x_{ij}+0.8)(0.5x_{i5}+0.5)) \le WS_0 \quad \text{(s32)}$$

$$\sum_{i=1}^{m}\sum_{j=1}^{n} R_{e,j}^+ L_{ij}(x_{ij} - x_{ij}') + \sum_{t=s+1}^{T} R_f^+ Ax_t + \lambda^- R_w \sum_{s=1}^{S} m_i^- A(x_t' - x_t) \le \frac{WT^-}{m_c^+} R_c^- \xi \quad \text{(s33)}$$

Then the result of the benefits of the water rights transfer is in the interval $(F^-(X), F^+(X))$, and the corresponding result of the decision variables is $X = ((x_s^-, x_s^+), (x_t^-, x_t^+), (x_{ij}^-, x_{ij}^+))^T$.

References in supplementary information

s1. Zhang, H. & Huang, F. *The exploration of water-saving potential and the theory of water rights transfer in the mainstream of the Yellow River*. (ed.Tian L.) 55-84 (The Yellow River Water Conservancy Press, 2009).

s2. Ningxia Water Conservancy. *The Report of Comprehensive Planning of Water Rights Transfer of the Yellow River in Ningxia* (ed. Feng P. et al.) 23-38 (Ningxia Water Conservancy, 2005).

s3. Ren, L., Sun, H. & Zhang, J. Analysis of hydrogeologic parameters for east irrigated areas of Qingtong Gorge of Ningxia. *Gansu Water Resour. Hydrop. Eng.* **36**, 281-283 (2000).

s4. Gao, Z., Gu, J. & Xu, J. Study on hydrologic and hydrogeologic parameters for west irrigated areas of Qingtong Gorge of Ningxia *Water Resour. Protect.* **19**, 14-16, doi: 10.3969/j.issn.1004-6933.2003.02.005 (2003).
